# Understanding the clinical manifestations of 16p11.2 deletion syndrome: a series of developmental case reports in children

**DOI:** 10.1097/YPG.0000000000000259

**Published:** 2020-07-22

**Authors:** Rana Fetit, David J. Price, Stephen M. Lawrie, Mandy Johnstone

**Affiliations:** a Simons Initiative for the Developing Brain, Centre for Discovery Brain Sciences, University of Edinburgh, Hugh Robson Building, George Square; b Division of Psychiatry, Centre for Clinical Brain Sciences, University of Edinburgh, Edinburgh, UK

**Keywords:** autism spectrum disorder, case reports, DNA copy number variations, 16p11.2 deletion syndrome

## Abstract

Copy number variants (CNVs) are genetic rearrangements, such as deletions and duplications, which result in a deviation from the normal number of copies of a given gene segment. CNVs are implicated in many neuropsychiatric disorders. Deletions of the human chromosomal region 16p11.2 are one of the most common genetic linkages to autism spectrum disorders (ASD). However, ASD is not the only presenting feature, and many patients with 16p11.2 deletions present with a variable clinical spectrum.

**Methods:**

To better understand the nature and presentation of the syndrome throughout development, we present three different, unrelated clinical cases of children with 16p11.2 deletion and provide a detailed description of their clinical manifestations.

**Results:**

Cognitive and motor impairments were characteristic of all three patients with 16p11.2 deletion, despite the differences in the extent and clinical presentation of impairment. Two patients had a clinical diagnosis of ASD and one showed several ASD traits. In addition, two patients also had severe speech and language impairments, which is in line with previous reports on 16p11.2 phenotypes. Although epilepsy and obesity have been frequently associated with 16p11.2 deletion, only one patient had a diagnosis of epilepsy and none of the three cases were obese.

**Conclusion:**

This variation in clinical phenotype renders correct clinical interpretation and diagnosis challenging. Therefore, it is critical to elucidate the variable clinical phenotypes of rare CNVs, including 16p11.2 deletions, to help guide clinical monitoring and counselling of patients and families.

## Introduction

Copy number variants (CNVs) represent recurrent chromosomal abnormalities and are associated with various phenotypic features (Lupski, 1998; Shinawi *et al.*, 2010; Jensen and Girirajan, 2019). Chromosome 16 is particularly rich in highly homologous low-copy repeats (LCRs), that mediate such genetic rearrangements via LCR-mediated nonallelic homologous recombination events during meiosis (Loftus *et al.*, 1999; Stankiewicz and Lupski, 2002; Martin *et al.*, 2004). Deletions of the human chromosomal region 16p11.2 are one of the most common genetic linkages to autism spectrum disorders (ASD) and account for approximately 1% of the cases (Kumar *et al.*, 2008; Weiss *et al.*, 2008; Fernandez *et al.*, 2010). Indeed patients with duplications of 16p11.2 appear to have a similar odds ratio to developing ASD as 16p11.2 deletions but interestingly the duplication carriers have a higher odds ratio of developing attention deficit hyperactivity disorder and a higher frequency of developing overall psychiatric disorders and psychosis (Niarchou *et al.*, 2019). However, ASD is not the only presenting feature in many patients with 16p11.2 deletion and only 25% of individuals with the deletion exhibit an autism phenotype (Hanson *et al*.; Jensen and Girirajan, 2019). 16p11.2 deletions are associated with a variable clinical spectrum of neurocognitive phenotypes and many other patients may manifest intellectual disability, morbid obesity, large head circumference, or epilepsy at varying degrees of penetrance (Bijlsma *et al.*, 2009; Bochukova *et al.*, 2010; Shinawi *et al.*, 2010). Even unaffected carriers of these CNVs are associated with cognitive deficits which may be subtle but confer significant disadvantages in educational attainment and ability to earn income in adulthood (Kendall *et al.*, 2019) as well as resulting in profound effects on physical health and mortality, even in those who have less profound early neurodevelopmental consequences (Crawford *et al.*, 2019). Early identification is therefore vital to allow appropriate medical and neuropsychiatric screening, support and treatment throughout the lifespan.

As with other microdeletion syndromes, the clinical heterogeneity and incomplete penetrance in 16p11.2 patients are quite remarkable which renders correct clinical interpretation and diagnosis challenging. To provide patients with a better clinical experience and ensure an enhanced developmental outcome, 16p11.2 deletion cases should be thoroughly phenotyped clinically. Several studies have presented a detailed phenotypic characterisation of individuals with 16p11.2 imbalances at the time of diagnosis revealing a variable clinical spectrum of neurocognitive phenotypes which included macrocephaly, developmental and language delay, cognitive impairment, as well as seizures (Fernandez *et al.*, 2010; Hanson *et al.*, 2010; Shinawi *et al.*, 2010). Another study has provided an elaborate description of the neurologic phenotypes of 16p11.2 deletion cases via comprehensive neurologic evaluation showing that despite the overlap with other neurodevelopmental disorders, speech and motor impairments, growth abnormalities, and tremors are striking features of 16p11.2 deletions that clinicians should be aware of (Steinman *et al.*, 2016). Here, we present three different, unrelated clinical cases of children with 16p11.2 deletion and provide a detailed description of their clinical manifestations from birth and throughout development. Despite the limited number of cases described here, presenting the complete developmental history and progression of the symptoms will help better understand the nature and presentation of the syndrome throughout development and thereby assist in accurate clinical diagnosis and early interventions.

## Methods

This study was approved by the School of Philosophy, Psychology and Language Sciences (PPLS) Research Ethics Committee of the University of Edinburgh (reference number 286-1819/2). Participants were recruited through online platforms, such as Simons Variation in Individuals Project (Simons VIP) connect social group. A thorough explanation of the study was provided to the guardians of the three children, and they gave their informed consent to participate. Parents were then interviewed in person or through video chat (example: Skype) if they were not residents of the UK, and were asked questions on (1) the clinical symptoms of their children throughout development, (2) any medications being taken, (3) any relevant family history and finally (4) social/clinical measures taken by parent/guardian since diagnosis with 16p11.2 deletion.

## Results

### Case 1

An 11-year-old girl presented with a diagnosis of hypermobility, memory retention difficulties (both short-term and long-term), social difficulties and epilepsy. At birth, she was reported to have a large head circumference. At 6 months of age, she had her first epileptic seizure and continued to have episodes every other month until she was diagnosed with generalised idiopathic tonic-clonic epilepsy. At 13 months, she was administered sodium valproate (Epilim) for the treatment of epilepsy. At 16 months, her parent started noticing signs of impaired mobility, such as lack of balance and frequent falling. By 3 years of age, her valproate medication was stopped because she had stopped having seizures but this resulted in a longer seizure that kept reoccurring over a period of 7 h. She was treated with phenytoin and put back on her former medication.

At school, this patient started showing signs of delayed learning and impaired memory retention as well as weight fluctuations. Symptoms of impaired mobility (imbalance, inward toeing while walking and continuous falling) still persisted and at 8 years old, she was diagnosed with hypermobility. By the age of 9, she was referred to an endocrinologist who offered genetic testing. Array comparative genomic hybridisation revealed a deletion in the proximal region of the genomic locus 16p11.2 (29 673 953–30 198 600), spanning approximately 524 kb. At this time, the patient continues to have moderate–severe learning and memory impairments. She is also presented with several ASD traits, although no official ASD diagnosis has been made. For example, she struggles with change of routine and gets emotionally attached to objects. Moreover, she has sensory difficulties. She dislikes loud noises, music or over-populated areas. The patient also struggles with social nuance and social cues. According to her parent, she has a very low opinion of herself and struggles to regulate her emotions. Her parent also continues to notice absence seizures, but no official diagnosis has been made.

### Case 2

A 5-year-old girl presented with a diagnosis of ASD, developmental delay, speech delay and anxiety. The mother was reported to have complications during pregnancy. She haemorrhaged the night before her due date and the following afternoon she was induced. At birth, the child had low birth weight (2.88 kg) and was supported in a temperature-controlled incubator. At the age of 3, her parents started noticing signs of speech and motor delay; however, a diagnosis of ASD was not met at this stage. She was then supported by an occupational therapist, to develop and strengthen fine and gross motor skills. However, at 4 years, she was diagnosed with ASD, global delay and anxiety and was offered genetic testing by her paediatrician. Molecular karyotyping results revealed a deletion in chromosome 16, region 16p11.2 (29 634 212–30 199 805). This spans approximately 565 kb. Currently, she is not on any medications. Her parents have taken environmental measures to ensure appropriate lighting and noise and provided her with weighted and sensory toys. The patient continues to show speech and motor impairments, with no reports of seizures.

### Case 3

An 8-year-old boy presented with a diagnosis of ASD together with childhood apraxia of speech. He was born with low birth weight (2.60 kg) and slight macrocephaly. From 3 to 6 months, he kept losing weight. Between 6 and 10 months, he was diagnosed with rickets and was given vitamin D supplements. By the age of 2, the patient showed symptoms of social and emotional delay and was offered occupational therapy, physical therapy and speech therapy. He was then referred to a neurologist who diagnosed him with ASD around the age of 3. The patient also showed severe language impairments, whereby the age of 3 he could only speak three words and communicated with his parents through sign language for 2 years. He was also reported to have a few episodes of febrile seizures; however, EEG tests showed no signs of epilepsy and seizures resolved during childhood. Following that, he was offered genetic testing by his paediatrician. Chromosomal microarray analysis revealed a deletion in the short arm of chromosome 16; particularly at the region 16p11.2 (29 567 295–30 177 916) which spans approximately 611 kb.

Between 4 and 5 years, the patient started exhibiting repetitive movements as well as verbal/phonetic tics triggered by calling his name. This included foot rubbing to full leg rubbing, refusing to walk on lines and not being able to walk through light beams shining through a window. Currently, he is not on any medications. He has a poor muscle tone, slight scoliosis and continues to show speech and language impairments.

## Discussion

Recurrent 16p11.2 deletions are characterised by a spectrum of neurocognitive phenotypes that are variably expressed amongst patients. We describe herein the phenotypes of three subjects with the deletion of the genomic region within chromosome 16p11.2. Table 1 provide a summary of the different clinical manifestations and clinical data of the patients together with the genetic locus of the deletions. The effects of 16p11.2 deletion on mean intelligence quotient have been shown to be a decrease of approximately 2 SDs (D’Angelo *et al.*, 2016; Hippolyte *et al.*, 2016) and intellectual disability is more frequent amongst deletion carriers (Niarchou *et al.*, 2019). Similarly, 16p11.2 deletion carriers showed the worst performance in a series of cognitive tests and were associated with statistically significant reductions in the number of offspring, suggesting deficits in socialising and forming families, as a consequence of cognitive, medical and behavioural problems (Kendall *et al.*, 2019). Cognitive and motor impairments were characteristic of all three patients with 16p11.2 deletion, despite the differences in extent and clinical presentation of impairment. This is in line with the clinical findings of other studies on 16p11.2 deletion carriers (Hanson *et al.*, 2010, 2015; Shinawi *et al.*, 2010; Zufferey *et al.*, 2012). The 16p11.2 deletion has been repeatedly associated with ASD, and accounts for approximately 1% of ASD cases (Weiss *et al.*, 2008; Kumar *et al.*, 2008; Fernandez *et al.*, 2010). Moreover, ASD has been shown to be the second most prevalent diagnosis in 16p11.2 deletion carriers (Niarchou *et al.*, 2019). In this study, two patients had a clinical diagnosis of ASD and one showed several ASD traits. Two of the patients also had severe speech and language impairments, which have been reported in previous phenotypic characterisation of patients with 16p11.2 deletions (Shimojima *et al.*, 2009; Hanson *et al.*, 2010; Shinawi *et al.*, 2010; Schaaf *et al.*, 2011; Zufferey *et al.*, 2012). The 16p11.2 deletion region has also been associated with obesity (Kumar *et al.*, 2008; Bochukova *et al.*, 2010; Walters *et al.*, 2010), but none of the three cases described here were obese. On the other hand, two patients were reported to be born with very low birthweights and weight fluctuations during early childhood. Although epilepsy is the most frequent neurological disorder observed in 16p11.2 deletion carriers (Shimojima *et al.*, 2009; Hanson *et al.*, 2010; Shinawi *et al.*, 2010; Schaaf *et al.*, 2011; Zufferey *et al.*, 2012), only one patient had a diagnosis of epilepsy whereas the other two had no reports of seizures or seizures had resolved with age. Similarly, previous reports have shown that congenital anomalies, including vertebral and spinal-related anomalies (Zufferey *et al.*, 2012), to be strongly associated with deletion carriers, scoliosis was reported in only one patient.

**Table 1:** Summary of clinical data of the three cases throughout development

	**Case 1**	**Case 2**	**Case 3**
Premature birth	No (1 day before due date)	No (1 day after due date)	No
Birth weight	Average (3.30 kg)	Low (2.88 kg)	Low (2.60 kg)
Head circumference at birth	N/A^a^	33.3 cm	N/A^a^
Cognitive impairment	Yes: moderate-severe learning and memory impairments	Yes: global developmental delay, anxiety	Yes: social and emotional delay
Motor impairments	Yes: hypermobility, imbalance, inward-towing	Yes: motor delay	Yes: poor muscle tone
Language impairments	None	Yes: speech delay	Yes: childhood apraxia of speech
Childhood illnesses diagnosed	Hypermobility, memory retention and social difficulties, generalised idiopathic tonic-clonic epilepsy	ASD, developmental delay, speech delay and anxiety	ASD, childhood apraxia of speech, rickets
ASD diagnosis	No (but shows some traits)	Yes	Yes
Seizures	Yes: generalised idiopathic tonic-clonic epilepsy	None	Resolved: Febrile seizures
Medications	Sodium valproate (Epilim)	None	None
Age at genetic testing	9 years	4 years	3 year
Deletion locus	16p11.2 (29 673 953–30 198 600)	16p11.2 (29 634 212–30 199 805)	16p11.2 (29 567 295–30 177 916)

a N/A = measurements unavailable.

**Fig. 1:**
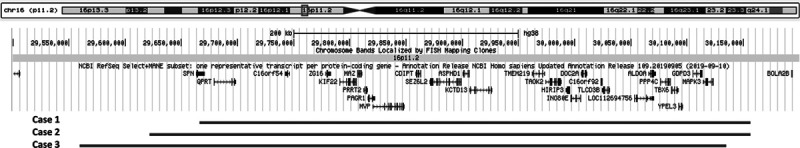
16p11.2 genomic locus. Red bars mark the approximate location of the deleted region of each case. Genes encompassed by the genomic are shown. Genomic positions are given according to the human genome build (GRCh38/hg38) assembly from the UCSC genome browser www.genome-euro.ucsc.edu.

All three patients had deletion in the proximal region of the 16p11.2 locus (Fig. 1). This genomic region spans around 600 kb and contains 47 genes, 28 of which are protein-coding. The flanking LCRs each span 147 kb and contain six duplicated genes, five of which are annotated as protein-coding (Weiss *et al.*, 2008; Jacquemont *et al.*, 2011; Tai *et al.*, 2016). NAHR-mediated CNV formation *in vivo* involves the mispairing of the flanking LCRs, which can result in either the loss or gain of a 740-kb segment equivalent to one copy of the 593-kb segment and one copy-equivalent of the LCR (Walters *et al.*, 2010). Several studies have demonstrated a dosage effect of 16p11.2 copy number on the various clinical findings, suggesting the presence of dosage-sensitive genes within the region (Weiss *et al.*, 2008; Shinawi *et al.*, 2010; Blumenthal *et al.*, 2014). Moreover, several genes within the 16p11.2 are promising candidates for the variable phenotypes reported in patients with the deletion. For example, MAPK3 is a synaptic signalling component which is reported to be necessary for several forms of learning (Mazzucchelli *et al.*, 2002; Pucilowska *et al.*, 2015) and quinolinate phosphoribosyltransferase encodes a key enzyme in catabolism of quinolinate, a potent endogenous exitotoxin to neurons, the elevation of which has been linked to the pathogenesis of epilepsy (Feldblum *et al.*, 1988; Haslinger *et al.*, 2018). Polymorphisms in the *TBX6* gene were associated with congenital scoliosis in the Han population (Wu *et al.*, 2015). Mice homozygous for a *TBX6* mutation were also reported to show rib and vertebral body anomalies (Watabe-Rudolph *et al.*, 2002), suggesting that *TBX6* is a candidate gene for vertebral malformations. On a molecular level, knockdown and overexpression studies revealed the implications of several genes like *MAPK3*, *KCTD13*, *MVP* and *TAOK2* that play critical roles in the growth and proliferation of progenitor cells as well as neurite morphogenesis in the pathology of ASD (Golzio *et al.*, 2012; de Anda *et al.*, 2012; Pucilowska *et al.*, 2015). Although the functions of other genes in this CNV remain poorly defined, these studies suggest that the dysregulation of cell cycle, neuronal migration and cortical lamination is fundamental for the pathogenesis of neurodevelopmental disorders, including ASD, in the developing brain (Hanson *et al.*, 2010; de Anda *et al.*, 2012; Packer, 2016; Casanova, 2014). Recently, it has been shown by modelling the 16p11.2 deletion in *Drosophila melanogaster* that a complex interaction of CNV genes operates in conserved pathways to modulate expression of the phenotype, many suppressing or enhancing cell proliferation pathways and are enriched in a human brain-specific network, providing translational relevance in humans (Iyer *et al.*, 2018). It is suggested that this CNV has pleiotropic effects, given that it intersects multiple genes. Recently, significantly associated phenotypes and medical consequences to 16p11.2 deletion in adult carriers included a high incidence of diabetes, osteoarthritis, hypertension, as well as asthma, anaemia and renal problems, indicating the need for regular medical monitoring (Crawford *et al.*, 2019).

In conclusion, it is clear that 16p11.2 deletions are manifested in a wide range of clinical symptoms which generally include developmental and language delay, cognitive impairment, seizures and ASD. The variations in clinical phenotypes in patients with 16p11.2 deletion render correct clinical interpretation challenging and frequently results in delayed diagnosis. All three cases reported here were at least 3 years of age before they received genetic testing, which highlights the importance of establishing and implementing early routine diagnostic testing so that functional and physical health outcomes can be addressed and treated at the earliest opportunity to reduce the likelihood of longer-term sequalae. As 16p11.2 deletion cannot be identified solely on the basis of clinical history, genetic testing should be a routine test in patients with neurodevelopmental disorders showing symptoms of speech and language delay, intellectual disability, or a diagnosis of ASD. Therefore, elucidating the variable clinical phenotypes of rare CNVs, including 16p11.2 deletions, remains critical to help guide clinical monitoring and counselling of patients and families.

## Acknowledgements

We would like to thank Simons VIP connect social group; an online community, funded by Simons foundation, that supports families with rare genetic changes associated with autism and developmental delay that allowed for the recruitment of participants for the study. We would like to specially thank the parents and children who agreed to take part in this study.

This work was funded by the Wellcome Trust (grant number: 628TRN G32492). The funder was not involved in conducting the research or preparation/submission of the manuscript.

## Conflicts of interest

There are no conflicts of interest.

## References

[R1] Bijlsma EK Gijsbers AC Schuurs-Hoeijmakers JH van Haeringen A Fransen van de Putte DE Anderlid BM Extending the phenotype of recurrent rearrangements of 16p11.2: deletions in mentally retarded patients without autism and in normal individuals. Eur J Med Genet. 2009; 52:77–87 19306953 10.1016/j.ejmg.2009.03.006

[R2] Blumenthal I Ragavendran A Erdin S Klei L Sugathan A Guide JR Transcriptional consequences of 16p11.2 deletion and duplication in mouse cortex and multiplex autism families. Am J Hum Genet. 2014; 94:870–883 24906019 10.1016/j.ajhg.2014.05.004 PMC4121471

[R3] Bochukova EG Huang N Keogh J Henning E Purmann C Blaszczyk K Large, rare chromosomal deletions associated with severe early-onset obesity. Nature. 2010; 463:666–670 19966786 10.1038/nature08689 PMC3108883

[R4] Casanova MF Autism as a sequence: from heterochronic germinal cell divisions to abnormalities of cell migration and cortical dysplasias. Med Hypotheses. 2014; 83:32–38 24780284 10.1016/j.mehy.2014.04.014 PMC4070182

[R5] Crawford K Bracher-Smith M Owen D Kendall KM Rees E Pardiñas AF Medical consequences of pathogenic CNVs in adults: analysis of the UK Biobank. J Med Genet. 2019; 56:131–138 30343275 10.1136/jmedgenet-2018-105477

[R6] D’Angelo D Lebon S Chen Q Martin-Brevet S Snyder LG Hippolyte L; Cardiff University Experiences of Children With Copy Number Variants (ECHO) Study; 16p11.2 European Consortium; Simons Variation in Individuals Project (VIP) Consortium. Defining the effect of the 16p11.2 duplication on cognition, behavior, and medical comorbidities. JAMA Psychiatry. 2016; 73:20–30 26629640 10.1001/jamapsychiatry.2015.2123 PMC5894477

[R7] de Anda FC Rosario AL Durak O Tran T Gräff J Meletis K Autism spectrum disorder susceptibility gene TAOK2 affects basal dendrite formation in the neocortex. Nat Neurosci. 2012; 15:1022–1031 22683681 10.1038/nn.3141 PMC4017029

[R8] Feldblum S Rougier A Loiseau H Loiseau P Cohadon F Morselli PL Lloyd KG Quinolinic-phosphoribosyl transferase activity is decreased in epileptic human brain tissue. Epilepsia. 1988; 29:523–529 3409840 10.1111/j.1528-1157.1988.tb03756.x

[R9] Fernandez BA Roberts W Chung B Weksberg R Meyn S Szatmari P Phenotypic spectrum associated with de novo and inherited deletions and duplications at 16p11.2 in individuals ascertained for diagnosis of autism spectrum disorder. J Med Genet. 2010; 47:195–203 19755429 10.1136/jmg.2009.069369

[R10] Golzio C Willer J Talkowski ME Oh EC Taniguchi Y Jacquemont S KCTD13 is a major driver of mirrored neuroanatomical phenotypes of the 16p11.2 copy number variant. Nature. 2012; 485:363–367 22596160 10.1038/nature11091 PMC3366115

[R11] Hanson E Bernier R Porche K Jackson FI Goin-Kochel RP Snyder LG; Simons Variation in Individuals Project Consortium. The cognitive and behavioral phenotype of the 16p11.2 deletion in a clinically ascertained population. Biol Psychiatry. 2015; 77:785–793 25064419 10.1016/j.biopsych.2014.04.021 PMC5410712

[R12] Hanson E Nasir RH Fong A Lian A Hundley R Shen Y Cognitive and behavioral characterization of 16p11. 2 deletion syndrome. J Dev Behav Pediatr. 2010; 31:649–657 20613623 10.1097/DBP.0b013e3181ea50ed

[R13] Haslinger D Waltes R Yousaf A Lindlar S Schneider I Lim CK Loss of the Chr16p11.2 ASD candidate gene QPRT leads to aberrant neuronal differentiation in the SH-SY5Y neuronal cell model. Mol Autism. 2018; 9:56 30443311 10.1186/s13229-018-0239-z PMC6220561

[R14] Hippolyte L Maillard AM Rodriguez-Herreros B Pain A Martin-Brevet S Ferrari C; 16p11.2 European Consortium, Simons Variation in Individuals Project Consortium. The number of genomic copies at the 16p11.2 locus modulates language, verbal memory, and inhibition. Biol Psychiatry. 2016; 80:129–139 26742926 10.1016/j.biopsych.2015.10.021

[R15] Iyer J Singh MD Jensen M Patel P Pizzo L Huber E Pervasive genetic interactions modulate neurodevelopmental defects of the autism-associated 16p11.2 deletion in Drosophila melanogaster. Nat Commun. 2018; 9:2548 29959322 10.1038/s41467-018-04882-6 PMC6026208

[R16] Jacquemont S Reymond A Zufferey F Harewood L Walters RG Kutalik Z Mirror extreme BMI phenotypes associated with gene dosage at the chromosome 16p11.2 locus. Nature. 2011; 478:97–102 21881559 10.1038/nature10406 PMC3637175

[R17] Jensen M Girirajan S An interaction-based model for neuropsychiatric features of copy-number variants. Plos Genet. 2019; 15:e1007879 30653500 10.1371/journal.pgen.1007879 PMC6336245

[R18] Kendall KM Bracher-Smith M Fitzpatrick H Lynham A Rees E Escott-Price V Cognitive performance and functional outcomes of carriers of pathogenic copy number variants: analysis of the UK Biobank. Br J Psychiatry. 2019; 214:297–304 30767844 10.1192/bjp.2018.301 PMC6520248

[R19] Kumar RA KaraMohamed S Sudi J Conrad DF Brune C Badner JA Recurrent 16p11.2 microdeletions in autism. Hum Mol Genet. 2008; 17:628–638 18156158 10.1093/hmg/ddm376

[R20] Loftus BJ Kim UJ Sneddon VP Kalush F Brandon R Fuhrmann J Genome duplications and other features in 12 Mb of DNA sequence from human chromosome 16p and 16q. Genomics. 1999; 60:295–308 10493829 10.1006/geno.1999.5927

[R21] Lupski JR Genomic disorders: structural features of the genome can lead to DNA rearrangements and human disease traits. Trends Genet. 1998; 14:417–422 9820031 10.1016/s0168-9525(98)01555-8

[R22] Martin J Han C Gordon LA Terry A Prabhakar S She X The sequence and analysis of duplication-rich human chromosome 16. Nature. 2004; 432:988–994 15616553 10.1038/nature03187

[R23] Mazzucchelli C Vantaggiato C Ciamei A Fasano S Pakhotin P Krezel W Knockout of ERK1 MAP kinase enhances synaptic plasticity in the striatum and facilitates striatal-mediated learning and memory. Neuron. 2002; 34:807–820 12062026 10.1016/s0896-6273(02)00716-x

[R24] Niarchou M Chawner SJRA Doherty JL Maillard AM Jacquemont S Chung WK Correction: psychiatric disorders in children with 16p11.2 deletion and duplication. Transl Psychiatry. 2019; 9:107 30837452 10.1038/s41398-019-0441-6 PMC6400999

[R25] Packer A Neocortical neurogenesis and the etiology of autism spectrum disorder. Neurosci Biobehav Rev. 2016; 64:185–195 26949225 10.1016/j.neubiorev.2016.03.002

[R26] Pucilowska J Vithayathil J Tavares EJ Kelly C Karlo JC Landreth GE The 16p11. 2 deletion mouse model of autism exhibits altered cortical progenitor proliferation and brain cytoarchitecture linked to the ERK MAPK pathway. J Neurosci. 2015; 35:3190–3200 25698753 10.1523/JNEUROSCI.4864-13.2015 PMC6605601

[R27] Schaaf CP Goin-Kochel RP Nowell KP Hunter JV Aleck KA Cox S Expanding the clinical spectrum of the 16p11. 2 chromosomal rearrangements: three patients with syringomyelia. Eur J Hum Genet. 2011; 19:152–156 20959866 10.1038/ejhg.2010.168 PMC3025795

[R28] Shimojima K Inoue T Fujii Y Ohno K Yamamoto T A familial 593-kb microdeletion of 16p11.2 associated with mental retardation and hemivertebrae. Eur J Med Genet. 2009; 52:433–435 19770079 10.1016/j.ejmg.2009.09.007

[R29] Shinawi M Liu P Kang SH Shen J Belmont JW Scott DA Recurrent reciprocal 16p11.2 rearrangements associated with global developmental delay, behavioural problems, dysmorphism, epilepsy, and abnormal head size. J Med Genet. 2010; 47:332–341 19914906 10.1136/jmg.2009.073015 PMC3158566

[R30] Stankiewicz P Lupski JR Genome architecture, rearrangements and genomic disorders. Trends Genet. 2002; 18:74–82 11818139 10.1016/s0168-9525(02)02592-1

[R31] Steinman KJ Spence SJ Ramocki MB Proud MB Kessler SK Marco EJ 16p11. 2 deletion and duplication: characterizing neurologic phenotypes in a large clinically ascertained cohort. Am J Med Genet A. 2016; 170:2943–2955 27410714 10.1002/ajmg.a.37820

[R32] Tai DJ Ragavendran A Manavalan P Stortchevoi A Seabra CM Erdin S Engineering microdeletions and microduplications by targeting segmental duplications with CRISPR. Nat Neurosci. 2016; 19:517–522 26829649 10.1038/nn.4235 PMC4903018

[R33] Walters RG Jacquemont S Valsesia A de Smith AJ Martinet D Andersson J A new highly penetrant form of obesity due to deletions on chromosome 16p11.2. Nature. 2010; 463:671–675 20130649 10.1038/nature08727 PMC2880448

[R34] Watabe-Rudolph M Schlautmann N Papaioannou VE Gossler A The mouse rib-vertebrae mutation is a hypomorphic Tbx6 allele. Mech Dev. 2002; 119:251–256 12464437 10.1016/s0925-4773(02)00394-5

[R35] Weiss LA Shen Y Korn JM Arking DE Miller DT Fossdal R Association between microdeletion and microduplication at 16p11. 2 and autism. New Engl J Med. 2008; 358:667–675 18184952 10.1056/NEJMoa075974

[R36] Wu N Ming X Xiao J Wu Z Chen X Shinawi M TBX6 null variants and a common hypomorphic allele in congenital scoliosis. N Engl J Med. 2015; 372:341–350 25564734 10.1056/NEJMoa1406829 PMC4326244

[R37] Zufferey F Sherr EH Beckmann ND Hanson E Maillard AM Hippolyte L; Simons VIP Consortium; 16p11.2 European Consortium. A 600 kb deletion syndrome at 16p11.2 leads to energy imbalance and neuropsychiatric disorders. J Med Genet. 2012; 49:660–668 23054248 10.1136/jmedgenet-2012-101203 PMC3494011

